# The challenge of removal of sepsis markers by continuous hemofiltration

**DOI:** 10.1186/s13054-019-2464-z

**Published:** 2019-05-15

**Authors:** Patrick M. Honore, David De Bels, Rachid Attou, Sebastien Redant, Kianoush Kashani

**Affiliations:** 1 ICU Department, Centre Hospitalier Universitaire Brugmann, Place Van Gehuchtenplein,4, 1020 Brussels, Belgium; 2 Division of Nephrology and Hypertension, Division of Pulmonary and Critical Care Medicine, Department of Medicine, Mayo Clinic, Rochester, USA

We enthusiastically read the paper by Elke et al. showing that mid-regional pro-adrenomedullin (MR-proADM) performance in the determination of the severity of illness and treatment response in sepsis to be more accurate in comparison with the established biomarkers such as C-reactive protein (CRP) and procalcitonin (PCT) [1]. We also like to highlight the validity of CRP, PCT, and MR-proADM measurements during continuous renal replacement therapy (CRRT). As Elke et al. indicated, CRRT is extensively used in sepsis (20.5% in the survivors and 58.1% in non-survivors). Acute phase reactant proteins (CRP and PCT) are effectively cleared by CRRT. CRP is predominantly present as a monomer (mCRP) in the blood [2] and is removed by all CRRT modalities given its low molecular weight (22–25 kDa) [2]. PCT is shown to be eliminated by convection, as well [3]. Also, dialysis membrane absorbs a substantial proportion of mCRP and PCT [3, 4]. As highly adsorptive dialysis membranes are increasingly used in CRRT worldwide, elimination of mCRP and PCT could be significantly accentuated. Hence, among CRRT patients, plasma levels of both biomarkers could be falsely low. On the other hand, the MR-proADM molecular weight (MW) is between 4 and 5.5 kDa, and, therefore, it may also be removed by CRRT. Indeed, Mueller et al. showed a significant decrease in MR-proADM (45–65%) if a high-flux membrane was used (with a cut-off of 35,000 Da) [4]. This cut-off is similar to what is used in contemporary CRRT membranes [5]. In Elke et al. study, 20.5% of the survivors and 58.1% of the non-survivors received CRRT, and the value of MR-proADM was 4.0 and 8.2 nmol/L in each group (*P* value < .001), respectively. As the average MR-proADM, CRP, and PCT levels in each group could have been impacted by differences in the CRRT prevalence, we recommend a sensitivity analysis to be done after the exclusion of CRRT patients to clarify the performance of these biomarkers when they are not artificially removed by an extracorporeal purification technique. At this point, there is no biomarker proven to reliably predict sepsis or infection among CRRT patients. Hence, the odyssey continues!

## Abbreviations

CRP: C-reactive protein
CRRT: Continuous renal replacement therapy
mCRP: Monomeric CRP
MR-proADM: Mid-regional pro-adrenomodullin
MW: Molecular weight
PCT: Procalcitonin
